# Mapping global trends in research of stem cell therapy for COVID-19: A bibliometric analysis

**DOI:** 10.3389/fpubh.2022.1016237

**Published:** 2022-10-14

**Authors:** Xinkang Zhang, Jiehui Cai, Lingzhi Chen, Qiuping Yang, Huiting Tian, Jinyao Wu, Zeqi Ji, Daitian Zheng, Zhiyang Li, Yexi Chen

**Affiliations:** Department of General Surgery, The Second Affiliated Hospital of Shantou University Medical College, Shantou, China

**Keywords:** COVID-19, stem cell, bibliometrics, VOSviewer, CiteSpace

## Abstract

Over the past 2 years, the world has witnessed the devastating effects of the COVID-19 pandemic on humanity. Fortunately, stem cell therapy is widely used in clinical practice for the treatment of COVID-19 and has saved the lives of many critically ill patients. A bibliometric analysis of this field can analyze research hotspots and predict the research trends. This research analyzed documents from Web of Science between the years 2020–2022. The bibliometrics software bibliometrix, VOSviewer, and CiteSpace were used to complete the visual analysis of publications, authors, countries, documents, organizations, collaborative networks, and keywords clustering. 896 publications on COVID-19 stem cell therapy were included in the analysis, including 451 articles and 445 review articles. The field grew at the average growth rate of 103.17% between 2020 and 2021. The United States had the highest number of publications and citations. Many developing countries had also contributed significantly to the field. The journal with the most articles was *Stem Cell Research and Therapy*. The most cited journal was *Stem Cell Reviews and Reports*. The published documents were focused on five themes: “Cell Biology”, “Medicine Research Experimental”, “Cell Tissue Engineering”, “Immunology”, and “Pharmacology Pharmacy”. The bibliometric analysis revealed that current clinical trials had validated stem cell therapy's remarkable potential in treating COVID-19 and its complications. It is foreseeable that future research in this area will continue to increase. With the help of bibliometric analysis, researchers can identify the current state of research and potential research hotspots.

## Introduction

Since the World Health Organization declared a pandemic on March 11, 2020, Coronavirus disease-19 (COVID-19), caused by the novel severe acute respiratory syndrome coronavirus 2 (SARS-CoV-2), has rapidly spread worldwide (1). As of August 24, 2022, a total of 593 million cases have been diagnosed on five continents, with more than 6.4 million deaths reported (2). These numbers continue to increase after multiple rounds of mass vaccination. There are various clinical manifestations of SARS-CoV-2, including mild to moderate respiratory depression, inflammation, hypoxia (3), infection of cardiomyocytes and cardiogenic shock (4), the tendency to clot in micro- and macrovascular vessels (5), myocarditis caused by the immune response (6), neurological and psychiatric disorders (7), and acute respiratory distress syndrome (ARDS) (8), which has high morbidity and mortality rates.

As a primary problem that endangers the health of all human beings, research on COVID-19 has become a common focus in more than 200 countries worldwide. At this stage, COVID-19 and the multiple complications it causes are still endangering patients' lives. New therapeutic approaches are urgently needed to attenuate the body's excessive immune and inflammatory response (9), accelerate lung function recovery and reduce mortality in patients with severe COVID-19. Stem cells have powerful immunomodulatory functions, and 80–90% of cells often accumulate in the lungs after administration (10). Stem cell therapies can be used to treat severe health conditions (11), such as mesenchymal stem cells therapy (MSCs therapy), which has been used in the treatment of influenza patients and animal models (12) to suppress chronic inflammatory responses through the release of anti-inflammatory and antifibrotic cytokines (13). Positive results have been demonstrated in studies including, but not limited to, asthma (14), acute lung injury (ALI) (15), and chronic obstructive pulmonary disease (COPD) (16). Cytokine storm is the most severe complication of COVID-19 (17). The human immune system is induced by COVID-19 to acutely release large amounts of pro-inflammatory cytokines such as IL-1, IL-6, and IFN-γ. Cytokine storm is inseparable from the progression of ARDS (18). Stem cells can effectively inhibit the release of inflammatory factors and subsequently reduce the probability of cytokine storm (19). Stem cells themselves can also secrete IL-10, cell growth factor, and Vascular endothelial growth factor (VEGF) to alleviate the symptoms of ARDS, effectively reduce pulmonary inflammatory infiltrates, repair damaged lung tissues, and reverse pulmonary fibrosis (20–22). So, with the ability of modulating immunity and suppressing inflammatory responses, stem cells can reduce the degree of tissue damage, which may be the main mechanism for the treatment of patients with COVID-19. Stem cell therapy, one of the most cutting-edge therapeutic approaches available today, a wide range of risks associated with SARS-CoV-2 infection can be reduced, and patients' lives can be saved.

However, to our knowledge, there is no bibliometric analysis specifically on COVID-19 stem cell therapy. Since the COVID-19 pandemic has not yet ended and treatment options for COVID-19 are still inconclusive, COVID-19 is still endangering the health and lives of patients with mild and severe diseases worldwide. Therefore, a bibliometric analysis of COVID-19 stem cell therapies is needed to clarify the current research state and inform future studies.

## Materials and methods

### Research method

First proposed by Pritchard (23), bibliometrics is widely used to analyze the disciplinary dynamics and research progress of a field. In bibliometrics, detailed information is obtained about the authors, countries, research institutions, journals, keywords, references, etc., to clarify the status of the research in a field of the literature (24). With the aid of modern computer technology, bibliometrics can visualize the analysis results in detailed graphs (25). It has been pointed out that visual analysis is a powerful supplement to the results, helping to interpret the data and help people better understand the results. At the same time, it can promote the exploration of deep links between documents.

### Data source and collection

We chose to search all literature on COVID-19 stem cell therapies from Thomson Reuters's Web of Science (WOS) core database, including editions of SCI-EXPANDED (2003–present), SSCI (2003–present), A&HCI (2003–present), ESCI (2015–present), CCR-EXPANDED (1985–present), and IC (1993–present). A major reason for choosing WOS was its comprehensive collection, which includes numerous biomedical studies, the high quality of the publications included and its recognition as the most comprehensive bibliometric database (26). To avoid bias in the results, we standardized the data. Specifically, different expressions of the same keywords were standardized to medical subject terms (Mesh), “Stem Cell”, and “COVID-19”, etc. The search query includes the following: #1, (((TS = (“Stem Cell^*^”)) OR TS = (“Progenitor Cell^*^”)) OR TS = (“Mother Cell^*^”)) OR TS = (“Colony-Forming Unit^*^”); #2, ((((((((((TS = (“COVID 19”)) OR TS = (“SARS-CoV-2 Infection^*^”)) OR TS = (“2019 Novel Coronavirus Disease^*^”)) OR TS = (“2019 Novel Coronavirus Infection^*^”)) OR TS = (“2019 nCoV Disease^*^ “)) OR TS = (“COVID 19 Virus Infection^*^”)) OR TS = (“Coronavirus Disease 2019”)) OR TS = (“SARS Coronavirus 2 Infection^*^”)) OR TS = (“COVID 19 Virus Disease^*^”)) OR TS = (“2019 nCoV Infection^*^”)) OR TS = (“COVID 19 Pandemic^*^”); #3, (TS = (Therap^*^)) OR TS = (Treatment^*^); #4, “#3” AND “#2” AND “#1”. The publications span from 2020 to 2022. The search of documents was completed on August 26, 2022, and a total of 979 articles were searched. Then we filtered the article type as “Article” and “Review Article” and the language as English and got 896 articles, including 451 articles and 445 review articles. A total of 83 articles were excluded, including 8 non-English articles, 1 non-English article review, 40 editorial materials, 25 letters, 8 meeting abstracts, and 1 correction. The retrieved papers were exported as plain text files with “full record and cited references”.

### Bibliometric analysis

We analyzed and organized the resulting documents, collecting their authors, titles, publications, affiliations, countries, abstracts, keywords, citations, etc. We chose to analyze the annual changes in publications using the following parameters. The average growth rate (AGR) represented the average of the annual growth rates and was calculated according to the following formula: AGR = [(Ending Value – Beginning Value)/Beginning Value] ^*^ 100. The compound average growth rate (CAGR) was the average annual growth rate over a specific period of more than 1 year. It was calculated using the following formula: CAGR = [(Ending Value/Beginning Value)^(1/n)^ – 1] ^*^ 100, where *n* was the number of years. The relative growth rate (RGR) was defined as the growth rate per unit of time, which was calculated according to the following formula: RGR = [(ln (S2) – ln (S1))/(t2 – t1)] ^*^ 100, where ln (S1) was the logarithm of the initial number of articles; ln (S2) was the logarithm of the final number of articles; t2 – t1 was the time difference between the final time and the initial time. The doubling time referred to the time it took to double the number of publications and was calculated using the following formula: doubling time = (t2 – t1) ^*^ lg2/(lgS1 – lgS2) (27, 28). The concepts of S1, S2, and t2 – t1 were the same as the above equation.

In the author analysis section, we chose the following formula to calculate the overall collaboration analysis for the COVID-19 stem cell therapy field: *C* = Nm/Nm + Ns. Here *C* referred to the degree of collaboration (DC); Nm referred to the number of multi-author papers; Ns referred to the number of single-author papers. The calculation formula for the collaboration index (CI) is the number of authors in multi-authored articles divided by the number of multi-authored articles (29).

We imported the data into VOSviewer, Biblioshiny (bibliometrix's web visualization interface), and Citespace for visual analysis and knowledge mapping. GraphPad Prism 9 (GraphPad Prism Software Inc., San Diego, CA, United States) was also used to create the required bar charts.

VOSviewer (30) version 1.6.18 (Leiden University, Leiden, Netherlands) uses a probabilistic-based approach to data normalization and is able to provide several types of visualization views. The meaning of the generated visualizations is based on three essential elements: size, distance, and color. Nodes represent specific objects such as keywords, countries, institutions, etc. The size of a node indicates the frequency of occurrence, with larger nodes indicating a higher frequency. The mutual distance between nodes indicates the closeness of the relationship. The smaller the distance, the closer the relationship between two nodes. And different colors represent different categories (30). We used it to construct co-authorship networks, coupling networks, co-citation networks, keyword co-occurrence networks, etc., and also analyzed the link strength between nodes (31).

Bibliometrix is an *R* tool that allows for the bibliometric analysis of data from WOS and the generation of accessible and engaging images (32). It is based on the *R* language version 4.1.2. With this software, we obtained several basic information, including the source of the literature, the number of references, and the contents of the literature. We move on to obtain further information, such as the average annual citation rate, the impact of authors and journals, the citation ranking of journals and articles, and the contribution of countries and organizations. The *h*-index is widely used to assess the number and applied impact of a researcher's scholarly output. If an author has *n* articles, each of which was cited at least *n* times among all scholarly articles, his *h*-index is *n* (33). Since the *h*-index is often limited by the number of publications and citations, it favors researchers with a certain number of years of experience. *G*-index is proposed as a supplement to the *h*-index. The papers are sorted in descending order by the number of citations, and the citations are stacked by serial number.

*G*-index is the final serial number such that the cumulative number of citations is no less then the square of the serial number (34).

This paper utilized CiteSpace (35) version 6.1.R2 to identify emergent keywords and references and to locate references and keywords associated with intense outbreaks.

**Figure 1:**
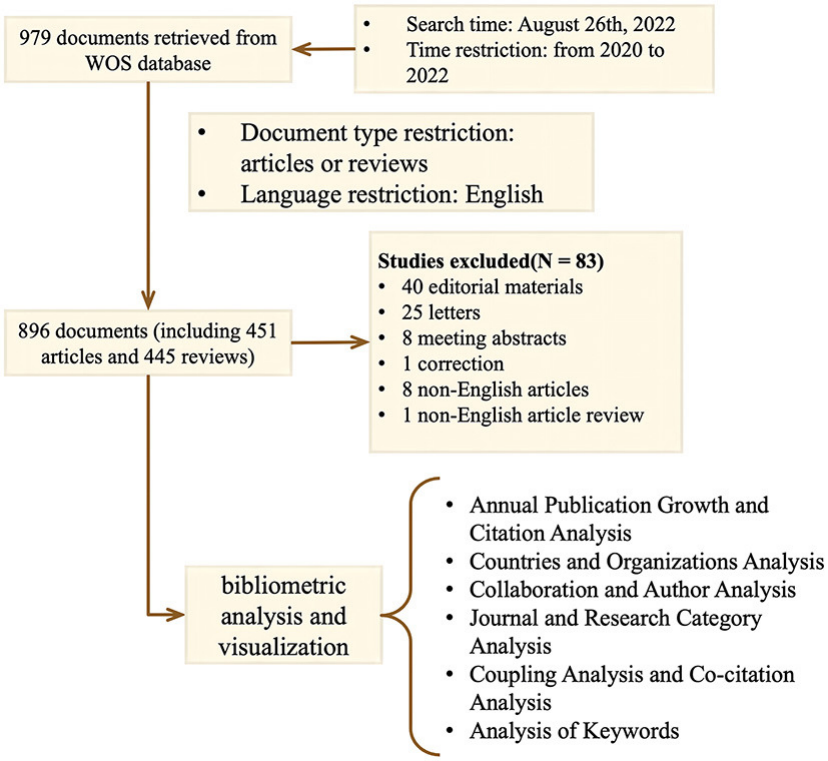
Flowchart of data collection and analysis.

Figure 1 shows the steps for screening the literature and analyzing the data in the form of a flow chart.

## Results

### Annual publication growth and citation analysis

The 896 papers selected for this study were written by 7,102 authors from 2,083 organizations in 97 countries and regions. They were published in 441 journals and cited a total of 49,629 references in 7,659 periodicals. The impact factor (IF) (36) was adopted from the latest annual Journal Citation Reports (JCRs) of 2022.

**Figure 2:**
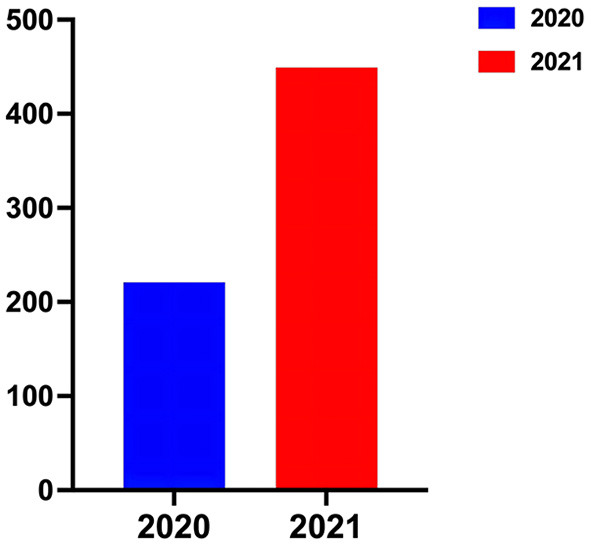
Bar chart of scientific production.

Since reports on COVID-19 first appeared in late 2019, research on COVID-19 stem cell therapies first appeared in 2020, with the average age of the articles at 1.02 years. As seen in Figure 2, 221 papers were published in 2020, accounting for 24.67% of the total. 449 articles were published in 2021, accounting for 50.11% of the total. Since 2022 was not yet closed, and the final issue volume is not yet known, the growth trend for that year could not be derived yet, so 2022 was not included in the analysis. From 2020 to 2021, the growth analysis shows the average growth rate (AGR) of 103.17%; the compound average growth rate (CAGR) of 103.17%; the relative growth rate (RGR) of 70.87%; and doubling time (DT) of 0.98 year. The high growth rate showed that the field was attracting considerable attention, and the number of studies continued to increase.

Citation analysis is a method of assessing the level of published journals, research institutions, and even researchers with the help of statistical methods. High-quality articles generally have a high number of citations (36). Table 1 lists the top 10 cited papers in the COVID-19 stem cell therapy field, and the highly cited papers indicate the research trends in the area. The most highly cited article in this field (37) was a systematic review with an IF38.637 score and 1,422 citations. It elucidated the significant potential of stem cell therapies to combat the most severe complication of COVID-19, cytokine storm (37), which was widely considered to be a direct cause of multiple fatal complications of COVID-19 (38). The second most cited article was “Transplantation of ACE2(-) Mesenchymal Stem Cells Improves the Outcome of Patients with COVID-19 Pneumonia” by Leng et al. (21). It was a clinical case study in which seven patients with COVID-19 injected with Mesenchymal stem cells (MSCs) showed improvement in lung function and symptoms of infection. No adverse effects were observed, and ACE2 (Angiotensin-converting enzyme 2), the primary receptor for COVID-19 (39), was used in the study to identify the presence of COVID-19 infection in MSCs injected into the body.

**Table 1:** Top 10 cited articles contributing to this research area.

**Documents**	**DOI**	**Total citations**	**TC per year**	**Normalized TC**
Ye et al. (37), J Infection	10.1016/j.jinf.2020.03.037	1422	474.00	28.87
Leng et al. (21), Aging Dis	10.14336/AD.2020.0228	658	219.33	13.36
Nishiga et al. (41), Nat Rev Cardiol	10.1038/s41569-020-0413-9	515	171.67	10.46
Li et al. (50), Int J Antimicrob Ag	10.1016/j.ijantimicag.2020.105951	476	158.67	9.67
Liu et al. (51), J Autoimmun	10.1016/j.jaut.2020.102452	404	134.67	8.20
Riva (52), Nature	10.1038/s41586-020-2577-1	356	118.67	7.23
Wang et al. (53), J Leukocyte Biol	10.1002/JLB.3COVR0520-272R	317	105.67	6.44
Lotfi et al. (54), Clin Chim Acta	10.1016/j.cca.2020.05.044	287	95.67	5.83
Sengupta et al. (44), Stem Cells Dev	10.1089/scd.2020.0080	263	87.67	5.34
Ulrich and Pillat (55), Stem Cell Rev Rep	10.1007/s12015-020-09976-7	235	78.33	4.77

From the top 10 cited articles, we can see that stem cell therapy, especially intravenous transplantation of MSCs, has an imperative role in treating critically ill patients and reducing cytokine storm. 6 of the top 10 articles were reviews, and 4 were clinical or scientific studies. Among all articles, 3 articles were cited more than 500 times, accounting for 0.33% of the total; 8 articles were cited more than 200 times, accounting for 0.89% of the total, and 32 articles were cited more than 100 times, accounting for 3.57% of the total.

### Countries and organizations analysis

**Figure 3:**
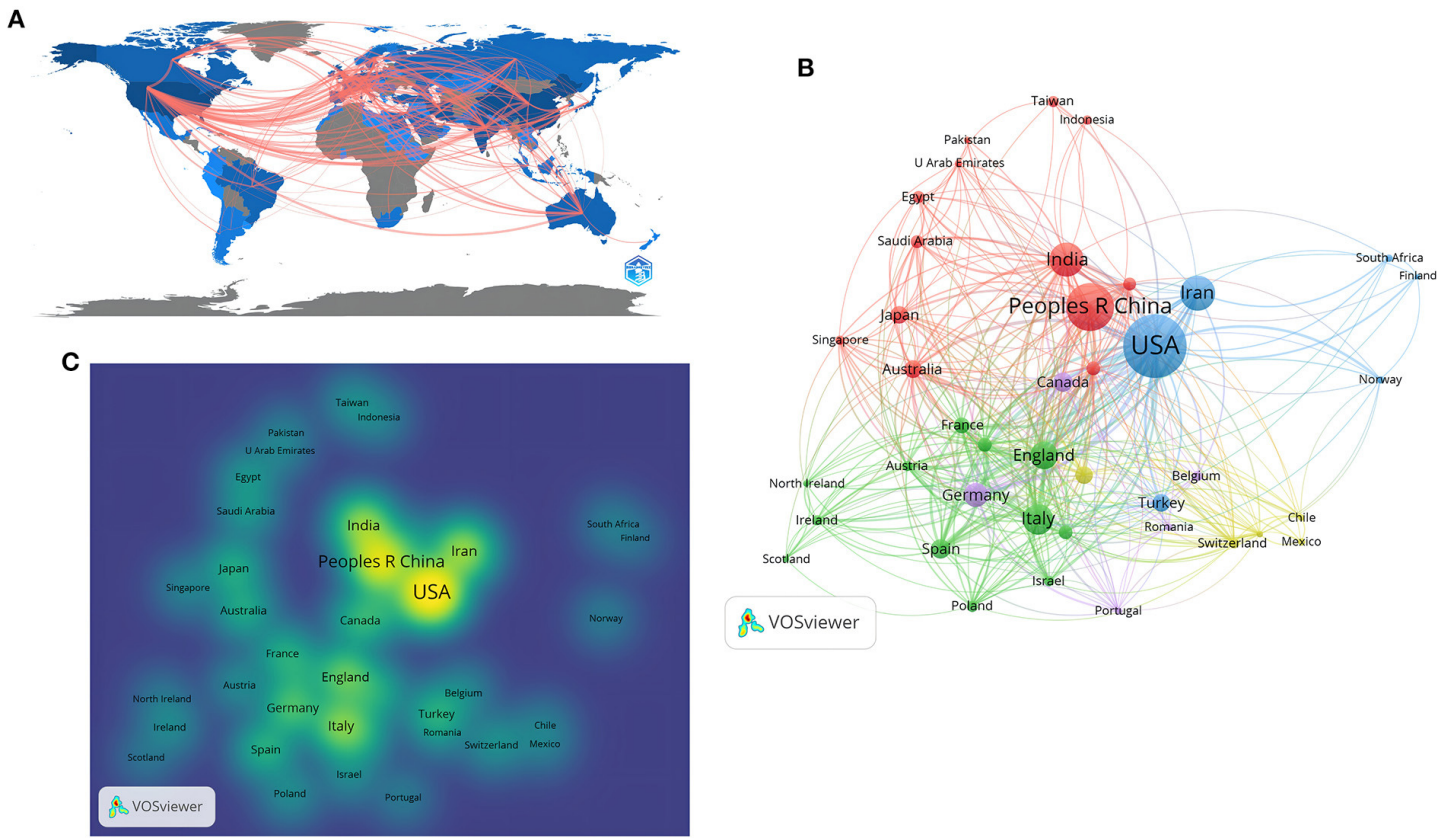
**(A)** Distribution and collaboration of country or region; **(B)** Network map of co-authorship between countries with more than six publications; **(C)** Density map of co-authorship between countries with more than six publications.

The focus on the treatment of patients with severe COVID-19 had attracted research contributions from 97 countries and regions and 2,083 organizations worldwide. As shown in Figure 3A, the main participating countries and regions are distributed in Europe, America, Asia, and Oceania. The darker the color means the higher the article output, and the denser connecting lines means the stronger partnership. The United States was the most published country in this field (*n* = 260, 29.02%), followed by China (*n* = 162, 18.08%), Iran (*n* = 97, 10.83%), India (*n* = 94, 10.49%), and Italy (*n* = 78, 8.71%) (Table 2). Using VOSviewer to plot Figure 3B, 41 countries meet the threshold of 5 articles as the minimum number of articles issued. More articles are produced at a larger node, and a closer distance represents a stronger relationship. It is divided into four clusters, each represented by a different color. In Figure 3C, the yellower colors represented more document output. The regional correlation showed that the U.S., China, India, and Iran issued the highest number of documents.

**Table 2:** Most productive countries contributing to this research field.

**Country**	**Documents**	**Citations**	**Average article citations**
USA	260	6,967	26.80
China	162	6,685	41.27
Iran	97	1,521	15.68
India	94	1,578	16.79
Italy	78	1,869	23.96
England	66	1,814	27.48
Germany	54	1,187	21.98
Spain	37	1,301	35.16
Canada	37	312	8.43
Japan	33	551	16.70

**Figure 4:**
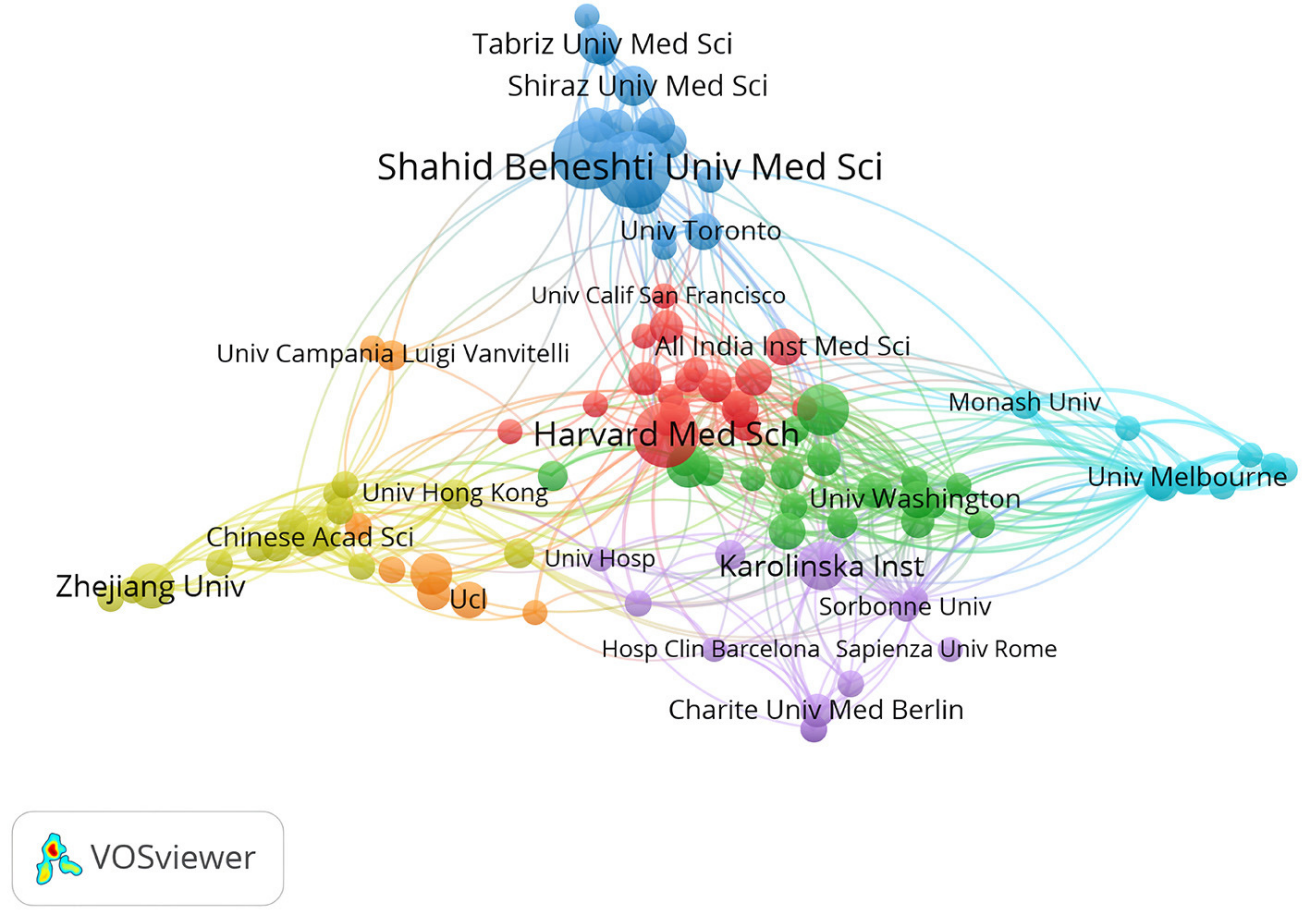
Network map of co-authorship between organizations in this field.

A total of 2,083 organizations were involved in research in this field, of which Shahid Beheshti University of Medical Sciences contributed the most papers (*n* = 33, 3.68%), followed by Tehran University of Medical Sciences (*n* = 28, 3.13%), Harvard Medical School (*n* = 25, 3.13%), Mayo Clinic (*n* = 17, 1.90%), Karolinska Institute (*n* = 15, 1.67%), and Zhejiang University (*n* = 14, 1.56%). It is worth noting that the average article citations of Zhejiang University reached 124.29, outperforming all organizations (Table 3). We then analyzed the co-authorship of 104 organizations with a threshold of 5 or more, divided into 7 clusters. The five organizations with the highest total link strength were Harvard Medical School (53), The Royal Melbourne Hospital (51), Peter MacCallum Cancer Center (50), Mayo Clinic (49), and Karolinska Institute (47) (Figure 4).

**Table 3:** Most productive organizations contributing to this research field.

**Organization**	**Documents**	**Citations**	**Average article citations**
Shahid Beheshti University of Medical Sciences	33	464	14.06
Tehran University of Medical Sciences	28	693	24.75
Harvard Medical School	25	1,112	44.48
Mayo Clinic	17	570	33.53
Karolinska Institute	15	253	16.87
Zhejiang University	14	1,740	124.29
Icahn School of Medicine at Mount Sinai	13	687	52.85
Huazhong University of Science and Technology	12	860	72.67
Shiraz University of Medical Sciences	11	50	4.55
Tabriz University of Medical Sciences	11	211	19.18

### Collaboration and author analysis

Based on our screened data, a total of 7,102 authors contributed to the field, 32 of whom published single-author articles. The average number of authors per article was 6.57. The average number of co-authors per article of 9.04, and an international co-authorship percentage of 30.17%. The degree of collaboration (DC) and the collaboration index (CI) values for each year from 2020 to 2022 were listed in Table 4, both increasing yearly. DC increased from 0.95 year in 2020 to 0.98 year in 2022, and CI increased from 8.08 in 2020 to 10.39 in 2022.

**Table 4:** The degree of collaboration (DC) and collaboration index (CI) in COVID-19 stem cell therapy.

**Year**	**Total number of authors**	**Number of single authored publications**	**Number of multi-authored publications**	**Number of authors in multi-authored publications**	**DC**	**CI**
2020	1,707	11	210	1,696	0.95	8.08
2021	3,706	17	432	3,689	0.96	8.54
2022	2,092	4	201	2,088	0.98	10.39

**Figure 5:**
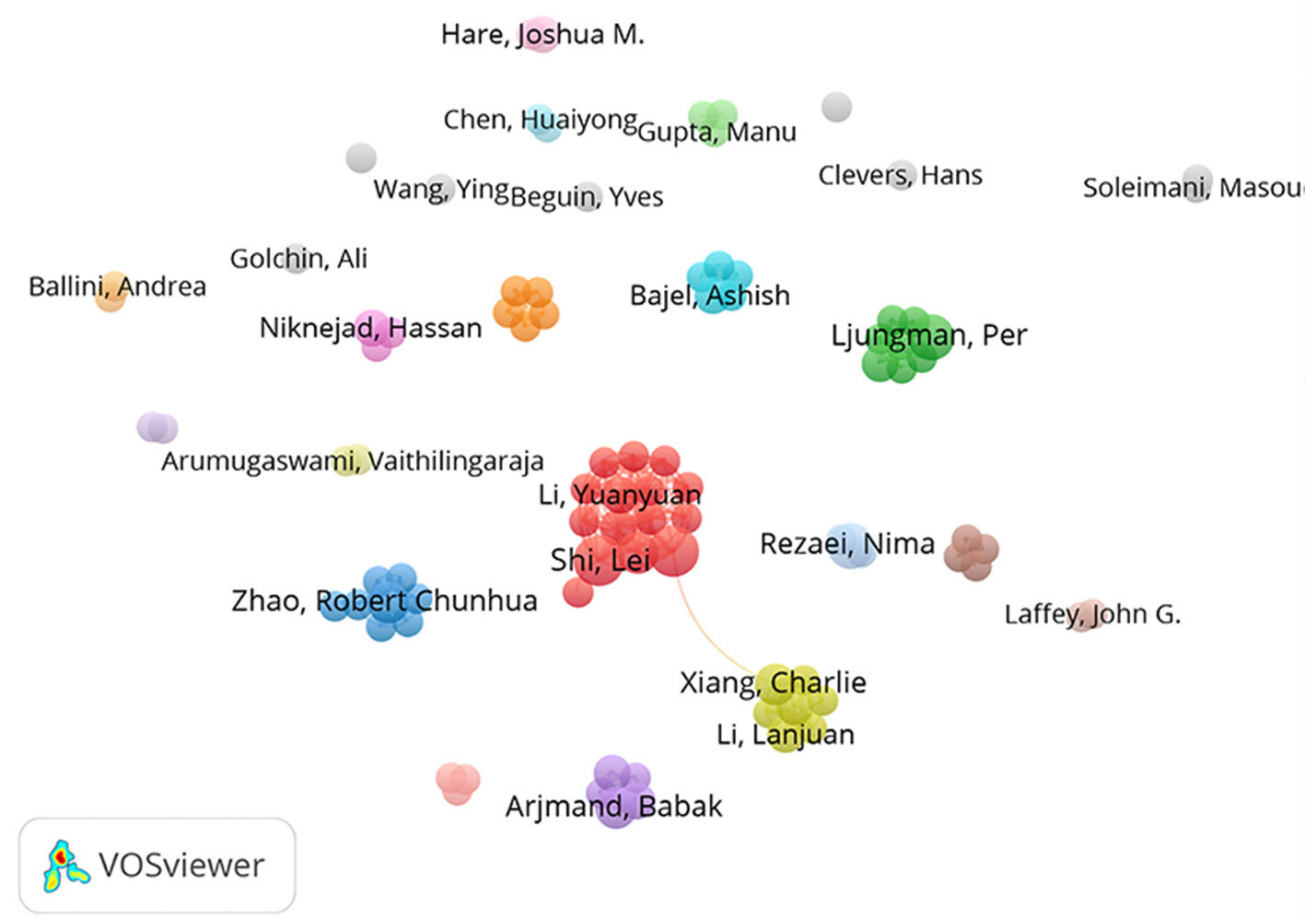
Network map of co-authorship of authors with four or more publications in this field.

The top 10 most prolific authors were listed in Table 5. Wang Fu sheng and Shi Lei were ranked first with 7 publications, accounting for 0.78% of the total publications, followed by Ljungman Per (*n* = 6, 0.67%), Zhao Robertchunhua, Xu Ruonan, Xiang Charlie, Arjmand Babak (*n* = 5, 0.59%). As seen in Figure 5, the 89 authors with three and more publications formed four larger co-author groups, with Huang Lei, Li Yuanyuan, Shi Lei et al. being the closest collaborators. The top five authors with the highest link strength were Wang Fu sheng (total link strength = 43), Shi Lei (total link strength = 42), Xu Ruonan (total link strength = 40), Zhang Chao (total link strength = 39), and Huang Lei (total link strength = 36). The number of citations was more indicative of the centrality of the authors due to the relatively short emergence of the field. Table 6 lists the top 5 authors with the highest number of citations, with Zhao Robertchunhua ranking first with 691 citations, followed by Rezaei Nima (396 citations), Shi Lei (369 citations), Wang Fu sheng (305 citations), and Xiang Charlie (283 citations).

**Table 5:** Most productive authors contributing to this research field.

**Authors**	**Documents**	**h-index**	**g-index**	**m-index**	**TC**	**NP**	**PY start**
Wang FS	7	5	7	1.667	305	7	2020
Shi L	7	5	5	1.667	369	5	2020
Ljungman P	6	5	6	1.667	196	6	2020
Zhao RC	5	4	4	1.333	691	4	2020
Xu RN	5	4	5	1.333	253	5	2020
Xiang C	5	4	5	1.333	283	5	2020
Arjmand B	5	2	2	0.667	14	2	2020
Hare JM	4	3	4	1.000	283	4	2020
Li YY	4	4	4	1.333	260	4	2020
Li LJ	4	4	5	1.333	242	5	2020

**Table 6:** Top 5 cited authors contributing to this field.

**Authors**	**Citations**	**h-index**	**g-index**	**m-index**	**NP**	**PY start**
Zhao RC	691	4	4	1.333	4	2020
Rezaei N	396	4	5	1.333	5	2020
Shi L	369	7	5	1.667	5	2020
Wang FS	305	5	7	1.667	7	2020
Xiang C	283	4	5	1.333	5	2020

The *h*-index is an important tool for evaluating the quantity and quality of academic output, especially in the medical field (40). On the *h*-index, Wang Fu sheng ranked highest (*h*-index = 6), while Das S, Ljungman Per, Shi Lei, Wang Fu sheng, and Zhang Robertchunhua ranked 2nd respectively (*h*-index = 5). The g-index is also considered a useful complement to the *h*-index if both researchers have identical *h*-index (34). Interpreting the result by referring to these two parameters will help identify the truly influential scholars. Of all the researchers, Wang Lu and Wang Fu sheng had the highest *g*-index (*g*-index = 7). Ljungman Per, Zhang Robertchunhua, and Kumar Santosh came second (*g*-index = 6), followed by Das S, Shi Lei, and Rezaei Nima (*g*-index = 5). Wang Fu sheng has the best output and quality in this field.

### Journal and research category analysis

The 896 papers in this dataset were published in 441 journals. The ten journals with the largest number of articles in this field are shown in Table 7. The top 10 journals published a total of 176 articles, accounting for 19.64% of the total number of publications. *Stem Cell Research and Therapy* had the most articles, totaling 33. *Stem Cell Reviews and Reports* ranked second, with 23 papers. The third was *Frontiers in Immunology*, with 25 papers. The following journals were *International Journal of Molecular Sciences and Cells*, with 20 and 18 papers, respectively. These journals are mainly focused on cell biology and immunology-related fields. In terms of citations, *Stem Cell Reviews and Reports* ranked first with 799 citations, and Aging *and Disease* ranked second with 743 citations. They were followed by *Stem Cell Research and Therapy* (574 citations), *Cell Stem Cell* (411 citations), and *Frontiers in Immunology* (374 citations). The *h*-index can be used to measure the impact of these journals. *Stem Cell Research and Therapy* and *Stem Cell Reviews and Reports* had the highest *h*-index of 12. *Stem Cells Translational Medicine, Cells, Frontiers in Immunology*, and *International Journal of Molecular Sciences* all had an *h*-index of 7, followed by *Aging and Disease* (*h*-index = 6) (Table 8).

**Table 7:** Top 10 most published sources.

**Source**	**Documents**
Stem cell research and therapy	33
Stem cell reviews and reports	23
Frontiers in immunology	21
International journal of molecular sciences	20
Cells	18
Stem cells translational medicine	16
World journal of stem cells	13
Transplantation and cellular therapy	12
Frontiers in cell and developmental biology	11
Aging and disease	9

**Table 8:** Top 10 highest-impact journals in this area.

**Source**	**h-index**	**g-index**	**m-index**	**TC**	**NP**	**PY start**
Stem cell research and therapy	12	23	4.000	574	23	2020
Stem cell reviews and reports	12	22	4.000	799	22	2020
Stem cells translational medicine	7	16	3.333	362	15	2020
Cells	7	12	2.333	155	13	2020
Frontiers in immunology	7	16	2.333	374	16	2020
International journal of molecular sciences	7	13	2.333	187	15	2020
Aging and disease	6	9	2.000	743	9	2020
Cell stem cell	5	5	1.667	411	5	2020
Frontiers in cell and developmental biology	5	7	1.667	59	10	2020
Frontiers in pharmacology	5	7	1.667	51	7	2020

In terms of research categories, a total of 86 research categories were included in this dataset. Table 9 lists the top five research categories and their number of papers: “Cell Biology” (206 publications), “Medicine Research Experimental” (165 publications), “Cell Tissue Engineering” (156 publications), “Immunology” (90 publications) and “Pharmacology Pharmacy” (89 publications). In terms of publishers, the top five publishers in terms of number of publications were “Elsevier” (174 publications), “Springer Nature” (153 publications), “Mdpi” (93 publications), “Frontier Media Sa” (74 publications), and “Wiley” (65 publications).

**Table 9:** Top 5 active research subject categories.

**Research subject category**	**Documents**
Cell biology	206
Medicine research experimental	165
Cell tissue engineering	156
Immunology	90
Pharmacology pharmacy	89

### Coupling analysis and co-citation analysis

**Figure 6:**
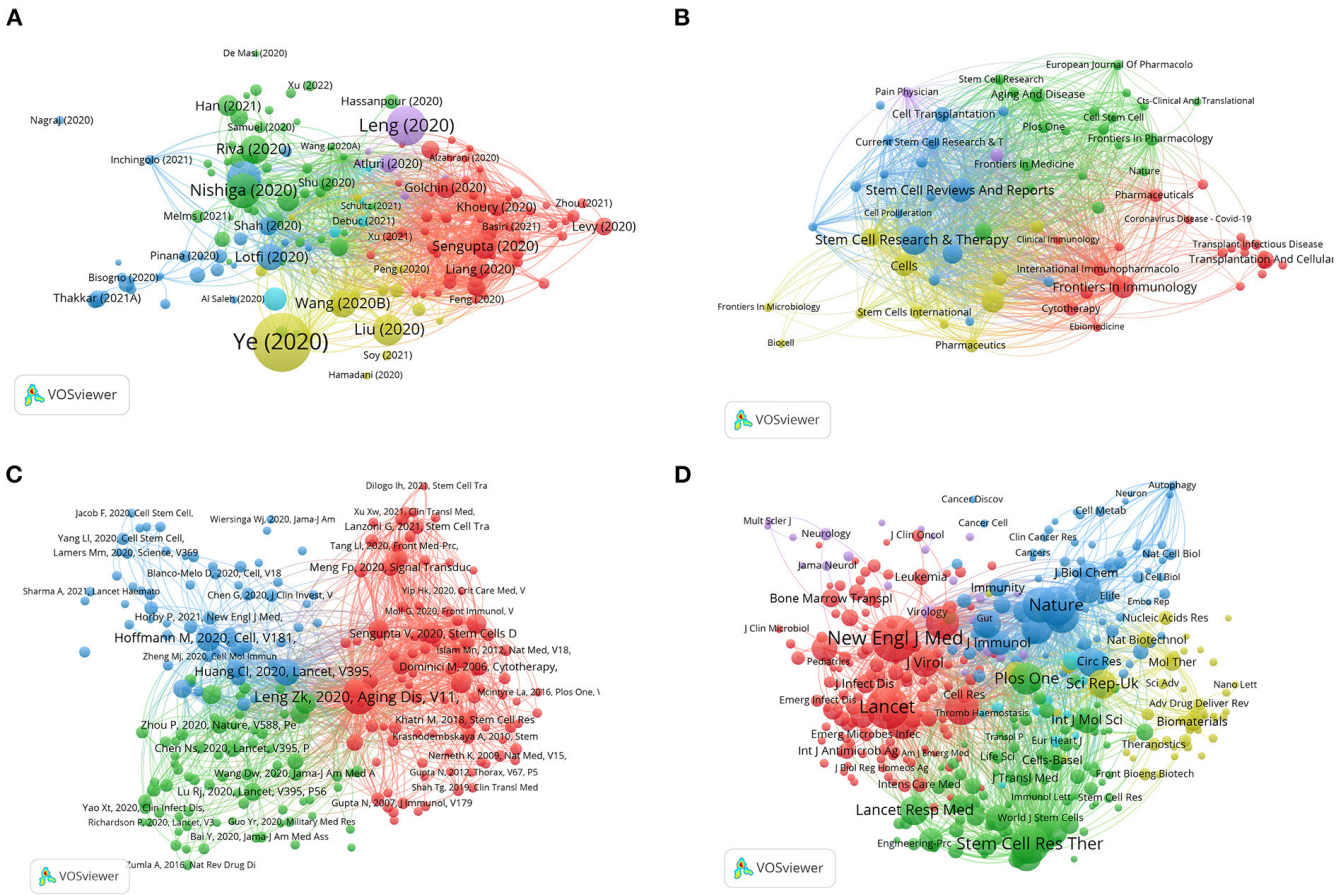
Bibliometric analysis of the bibliographic coupling and co-citation. **(A)** Bibliographic coupling of documents; **(B)** Bibliographic coupling of sources; **(C)** Co-citation of references; **(D)** Co-citation of sources.

**Figure 7:**
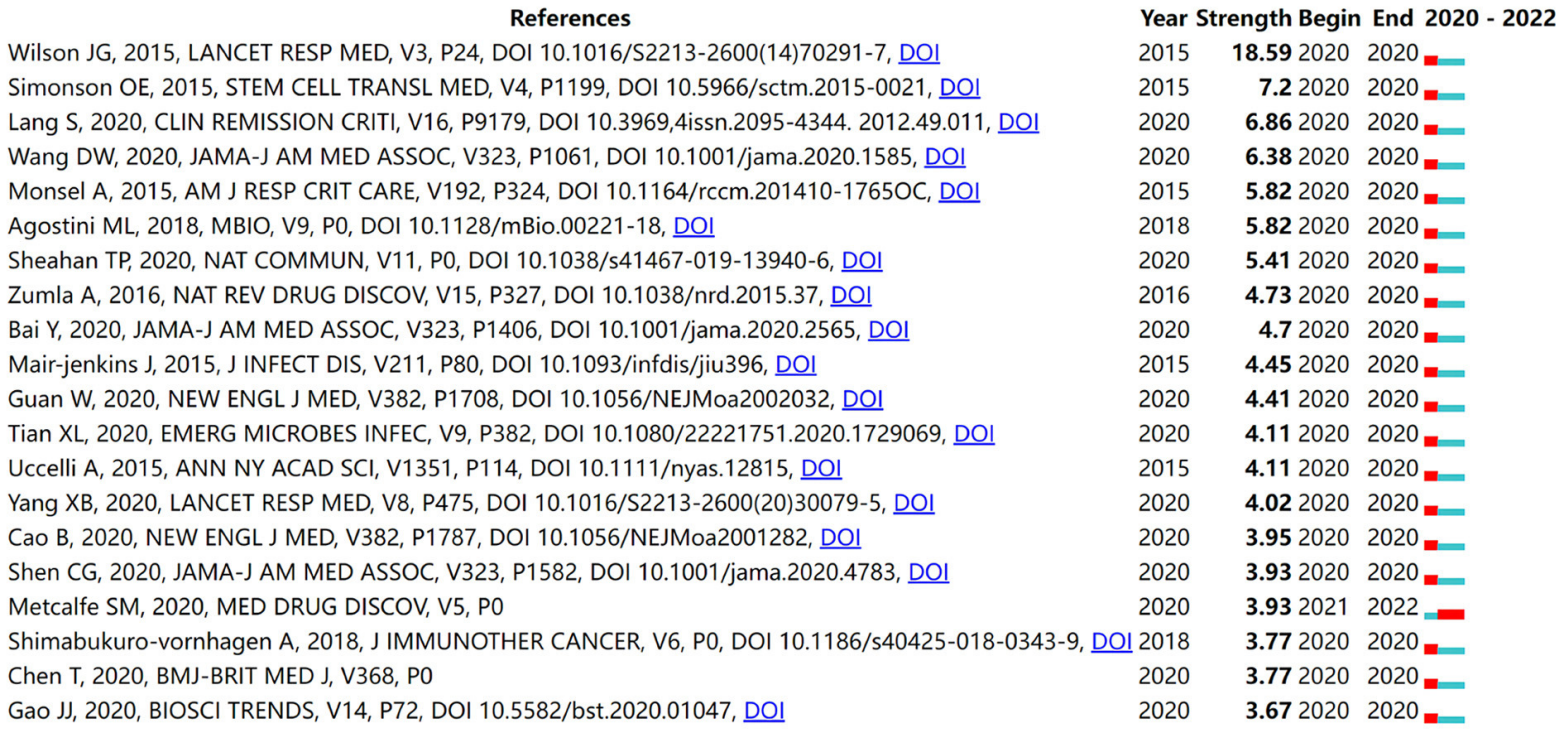
Top 20 articles with the strongest citation burst.

Figures 6A,B show the coupling analysis of literature and sources. Different colors represent different clusters. The node size represents the frequency of citations. In Figure 6A, using a minimum of 20 citations of a document as the threshold, a total of 152 articles were included in the analysis. They were divided into 6 clusters. The red cluster was the largest cluster and included 52 items. Its main themes were case analyses of COVID-19 stem cell therapy and phase reviews. Among other clusters, Ye et al.'s (37) “The pathogenesis and treatment of the “Cytokine Storm” in COVID-19”, Leng et al.'s (21) “Transplantation of ACE2—Mesenchymal Stem Cells Improves the Outcome of Patients with COVID-19 Pneumonia”, and Nishiga et al.'s (41) “COVID-19 and cardiovascular disease: from basic mechanisms to clinical perspectives” had the highest citation frequency. Using 20 citations of a cited reference as the threshold, four clusters of 228 references were obtained. The top cluster was indicated in red, with 97 objects representing the most influential research areas (Figure 6C). The top five most cited references were Leng et al. (21) (324 citations), Huang et al. (42) (207 citations), Hoffmann et al. (43) (180 citations), Sengupta et al. (44) (131 citations), Xu et al. (45) (126 citations). According to the co-citation analysis of article sources in Figure 6D, the largest cluster of citation sources included 113 items, including journals *The New England Journal of Medicine, Lancet, Blood*, and *JAMA*. The following journals were the most cited in the field: *The New England Journal of Medicine* (*n* = 1,786), *Lancet* (*n* = 1,540), *Nature* (*n* = 1,281), *Stem Cell Research & Therapy* (*n* = 1,215), and *Cell* (*n* = 1,074). Citation burst is an indicator used to detect the most active areas of research during different time periods. Using CiteSpace, we detected the 20 references with the strongest citation burst and generated the list (Figure 7). It was worth mentioning that the article with the highest citation burst intensity (strength = 18.82) by Wilson et al. (46), which documented a phase I clinical trial of MSCs for ARDS with favorable results. This was the second clinical trial of MSC worldwide, and all patients in this trial showed good tolerability. It was also worth mentioning that the paper “Mesenchymal stem cells and management of COVID-19 pneumonia” (47), which became a burst citation in 2021. It raised the problem that stem cells were difficult to be obtained and produced for large-scale clinical treatment and proposed the use of nanotechnology to synthesize LIF (leukemia inhibitory factor), the active ingredient produced by stem cells, for treatment.

### Analysis of keywords

**Figure 8:**
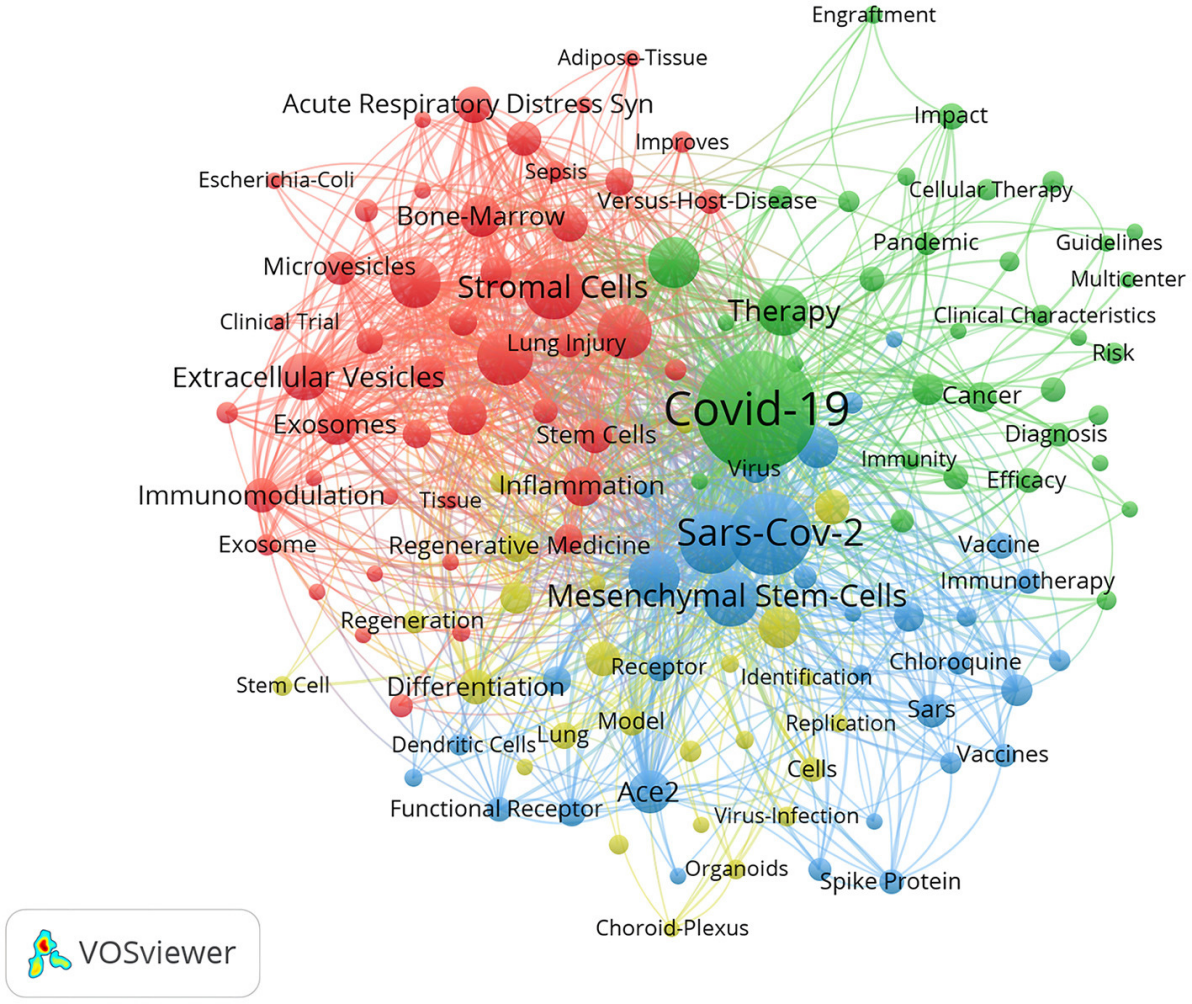
Network map of keywords with frequency more than ten.

The minimum threshold value of keyword occurrence was set to 10 times, and a total of 139 keywords were included in the analysis, generating a total of 4 clusters. Keywords with high relevance were merged into one cluster (Figure 8), and keyword clustering helped to find the focus of the research area. The red cluster contained the most keywords (*n* = 46), including “stromal cells”, “mesenchymal stem cell”, “acute lung injury”, etc. The green cluster was followed (*n* = 35), which includes “COVID-19”, “therapy”, “transplantation”, etc. Next was the blue cluster (*n* = 33), including “SARS-CoV-2”, “coronavirus”, “cytokine storm”, “ace2”, etc. Finally, the yellow cluster (*n* = 25) contained “infection”, “expression”, “regeneration”, and so on. Overall, the most frequently used keywords were “stromal cells” (*n* = 136), “mesenchymal stem-cells” (*n* = 115), “stem-cells” (*n* = 110), “coronavirus” (*n* = 94), “acute lung injury” (*n* = 93), etc.

**Figure 9:**
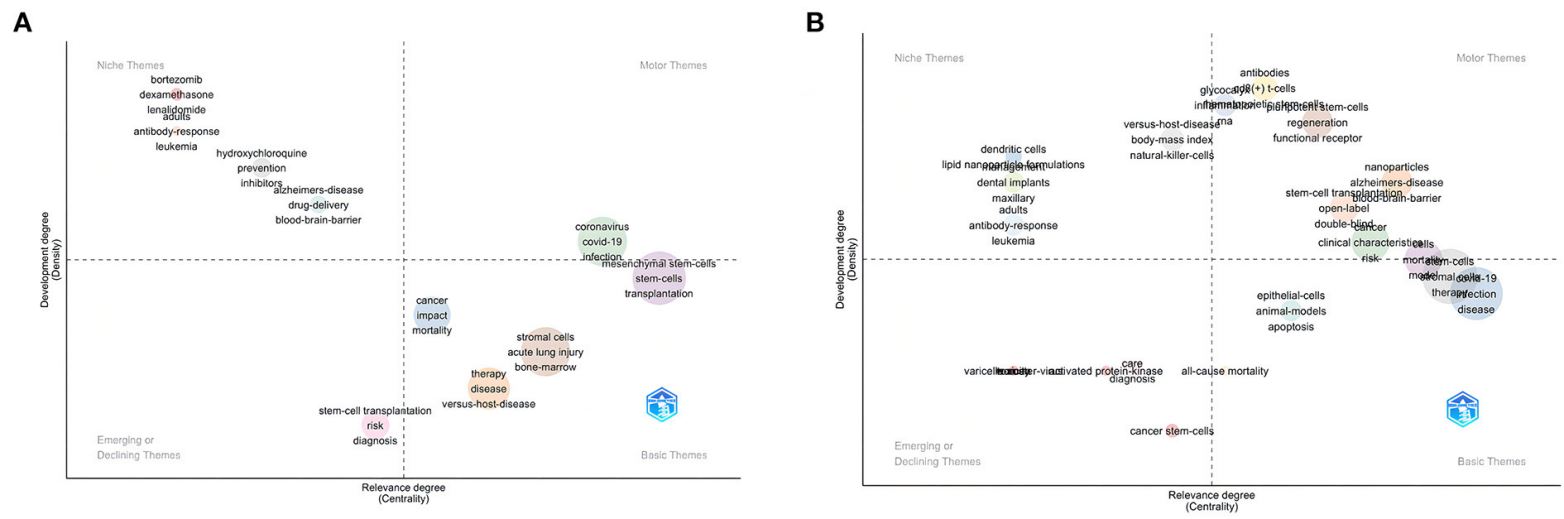
Keywords plus **(A)** Thematic map; **(B)** Thematic evolution map.

Keywords plus are those keywords that appear only in the titles of the references cited in the articles and do not appear in the titles of the articles (48). Figure 9A shows the thematic map drawn by analyzing keywords plus, divided into four quadrants in total. The horizontal axis is the Relevance degree (Centrality); the larger it is, the higher the relevance is. The vertical axis indicates the Development degree (Density); the larger it is, the more it is developed and studied. In addition, the first quadrant is Motor Themes, the topics in this quadrant have high centrality and high density, and are important topics in the current research field; the second quadrant is Niche Themes, in which the issues in this quadrant have high density but low centrality, meaning that they are highly developed but not very relevant to the research field and less critical; the third quadrant is Emerging or Declining Themes, the themes in this quadrant have low centrality and density, there are two possibilities for such themes, either they are being eliminated, or they are just emerging; as for the themes in the fourth quadrant, they have high centrality and low density, which means they have become the basic knowledge of the field. Figure 9A shows ten different categories of themes. The green clusters (keywords: “coronavirus”, “covid-19”, and “infection”) were Motor Themes in the domain, and the pink clusters (keywords: “stem-cell transplantation”, “risk”, and “diagnosis”) indicated Declining Themes. In addition, there are four clusters belonging to Basic Theme: orange cluster, including the keyword “therapy”, “disease”, and “versus-hot-disease”; brown cluster, including the keyword “stromal cells”, “acute lung injury”, and “bone-marrow”; and blue cluster, including the keyword “cancer”, “impact”, and “mortality”. Four clusters were belonging to Niche Themes. Figure 9B was the Thematic evolution map, which was used to predict the future changes of the clusters within Figure 9A. We can foresee great developments and changes in this field in the future. COVID-19 stem cell therapy research will give rise to more subdivided research directions. The emergence of more clusters in the first quadrant indicates that this research area holds great potential for growth. The emergence of keywords such as “antibodies” and “cd8(+) *t*-cells” reflects that the relationship between stem cell therapy and immunity will be an important research direction. Meanwhile, the increase of keywords such as “open-label”, “double-blind”, and “clinical characteristics” indicates more clinical trials in this field, which marks the further maturation of research.

**Figure 10:**
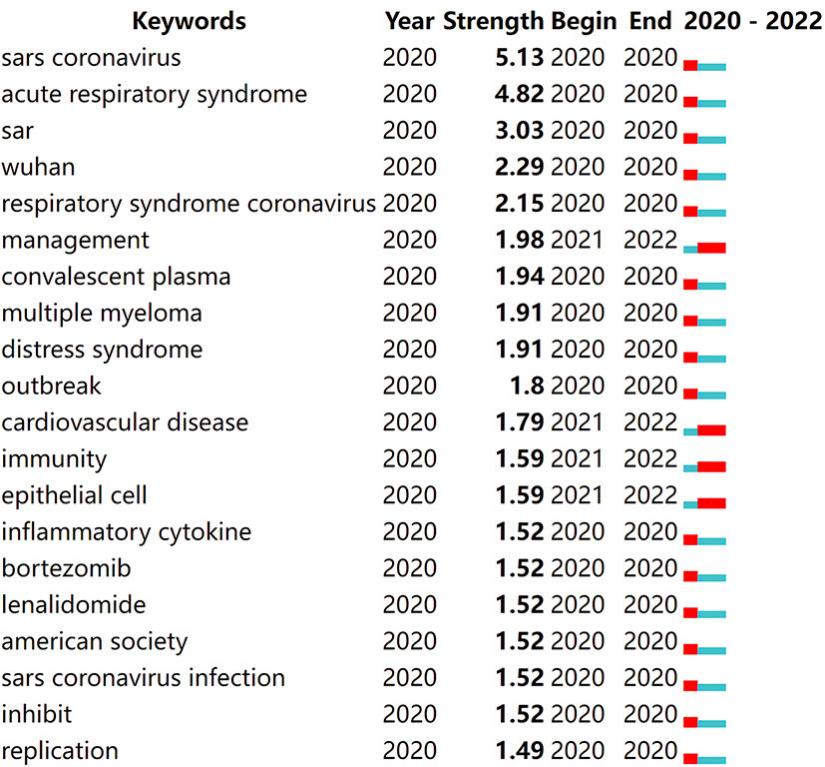
Top 20 keywords with the strongest keywords burst.

We used CiteSpace to detect words that frequently appear over time, i.e., burst keywords. Figure 10 shows the top 20 strongest burst keywords, most of whom became burst keywords in 2020. The five keywords with the highest outbreak intensity were almost pointed to sars coronavirus, which was because COVID-19, identified in the Chinese city of Wuhan, had so many similarities with SARS in clinical manifestations and at the molecular level, thus making it named SARS-CoV-2. This provided an opportunity to discover effective therapeutic drugs like those effective against SARS. “sars coronavirus” had the highest outbreak intensity at 5.13. “management”, “cardiovascular disease”, “immunity”, and “epithelial cell” became the burst keywords in 2021. They covered several aspects of COVID-19: pathogenic mechanism, clinical features, and treatment, indicating that researchers are committed to comprehensively understanding and controlling COVID-19 from basic theory to practical application, to better cope with this public health problem. The function of stem cells to regulate immunity has become a crucial aspect in the research of stem cell therapies because of their ability to inhibit the release of inflammatory cytokines and reduce the risk of developing multiple complications of COVID-19. The damage of alveolar epithelial cells by COVID-19 greatly affected the lung function of patients, and stem cells possessed the ability to differentiate into alveolar epithelial cells (49). Research in this area could effectively improve patient prognosis.

## Discussion

In this study, 896 publications in the field of COVID-19 embryonic stem cell therapy between 2020 and 2022 were selected for bibliometric analysis and visualization using software such as Bibliometrix, VOSviewer, and CiteSpace to get a more precise and more definitive picture of the latest research trends in the field. The number of articles published in this field has grown rapidly since 2020, with the compound average growth rate (ARG) of 103.17% and doubling time (DT) of 0.98 year, meaning that the number of articles doubled almost 1 year, signifying a booming field with great potential for growth. All articles included in the dataset were cited a total of 16,058 times, with an average of 17.92 citations per article. Six of the top 10 most cited papers are reviews (37, 50, 51, 53–55) summarizing the current clinical presentation of COVID-19, possible management options, and clinical manifestations and mechanisms of complications. The impressive results of stem cell therapy in the treatment of critically ill patients and the progress of current outcomes were also documented. In the most influential article, “The pathogenesis and treatment of the “Cytokine Storm” in COVID-19”, the COVID-19 infection was complicated by cytokine storm, resulting in ARDS and multiple infections. MSC regulated immunity to reduce the release of inflammatory factors and repaired damaged lung tissue (37). Among the most cited articles, Leng et al. reported that seven patients (One critically ill, four seriously ill, and two common patients) at Beijing Youan Hospital (Beijing, China) were injected with Mesenchymal Stem Cells (MSCs), and all showed improved clinical presentation after several days. In addition, MSCs were found to have an anti-inflammatory effect in COVID-19 patients and a decrease in key inflammatory markers (21). The paper also confirms that MSCs are ACE2- by 10x scRNA-seq survey for the first time. Considering that they act as receptors in the process of virus infection, this feature makes MSCs resistant to SARS-CoV-2 infection theoretically. The report by Wilson et al. (46), which documented the promising results and relatively reliable safety profile of MSCs in phase I clinical trial for the treatment of ARDS, provides a solid foundation for current research on the use of stem cell therapies in patients with COVID-19.

A total of 97 countries and regions were included in the study. The United States was the most prominent contributor to the field, with the most publications and citations. The contribution of developing countries in this field couldn't be ignored, with three of the top five countries. China ranked second in the number of published articles, while having the highest average number of citations per article (*n* = 41.27).

The authors in this field had high degree of collaboration (DC) and collaboration index (CI), and the trend was increasing year by year, indicating the strong collaboration of scholars in this field. Wang fu-sheng, Shi lei (*n* = 7, 0.78%), Ljungman per (*n* = 6, 0.67%), Zhao robertchunhua, Xu ruonan, Xiang charlie, Arjmand babak (*n* = 5, 0.59%) were the top 5 authors in this field in terms of publications, with Wang fu-sheng having the highest h-index and g-index. Zhao robertchunhua had the highest number of citations (691 citations), and Wang fu-sheng had the strongest link strength with other authors.

Seven of the top 10 journals with the highest number of articles belonged to “Cell Biology,” and the most cited journals were *Stem Cell Reviews and Reports* (799 citations), *Aging and Disease* (743 citations), *Stem Cell Research and Therapy* (574 citations), which shows the importance of cell-level research in the field.

There is no doubt that stem cell related keywords were critical, occupying the top positions in terms of frequency of occurrence. The following keyword was coronavirus and lung injury related, while the high frequency of “mesenchymal stem-cells” was closely associated with many clinical trials of MSCs injections, which had been shown to have the ability to modulate hyperimmune and inflammation and to alter the cytokine storm to improve the clinical condition of patients. In addition, MSCs themselves had been shown to be clinically effective against a range of lung diseases and reduce pulmonary fibrosis (22, 56).

Although intravenously infused MSCs have been shown to migrate into the lung and secrete a variety of factors that play an important role in modulating immunity, improving lung function, and combating pulmonary fibrosis, much of the research on stem cell therapy is still in the early stages of clinical trials (57–59). The large-scale application of stem cell therapy is still facing some obstacles at this stage due to insufficient research. First, the issue of stem cell heterogeneity is one of the most important problems in the clinical application of stem cell therapy for COVID-19. It is currently impossible to accurately guarantee the consistency of stem cell quality. In addition, the clinical efficacy of stem cell therapy is affected by many factors such as safety, cell viability, cell source, frequency and route of administration, etc. Current trial results are insufficient to cover the actual situation of hundreds of millions of infected patients worldwide, and the commonly used modalities may have unpredictable effects on a small number of patients. For example, intravenous stem cells are currently the most used route of administration in COVID-19 stem cell therapy. This is because intravenous stem cells can induce the retention of large numbers of stem cells in the lungs through pulmonary effects, which can promote lung tissue repair (60). However, some studies had found that intravenous stem cell administration to critically ill patients on ECOM caused cells to adhere to membrane oxygenator fibers, resulting in a significant reduction in blood flow through the membrane (61), so endotracheal administration is often more appropriate for this group of patients.

There are still significant limitations in our study. First, our data sources were limited to Web of Science (WOS). Due to the limitations of existing software technology, it was impossible to directly combine multiple databases' search results. The reason we chose to use WOS rather than other databases (e.g., Embase, PubMed, etc.) is that it is recognized as the most used and suitable bibliometric database in scientometrics, and its format can be directly recognized by the bibliometric software used in this paper. In addition, the language of the selected literature was limited to English, so some multilingual studies were not included. Finally, the literature selection criteria included in the research were subjective, so that not all the documents included in the analysis were in line with the requirements. Our findings apply only to the time point of our study (August 26, 2022), and we believe that our research will be of some help to future scientific studies.

## Conclusion

Therapies for treating COVID-19 by injecting stem cells have achieved the expected results in multiple clinical trials worldwide. Technology is still evolving rapidly, making them a powerful tool in the fight against high-risk diseases and emerging diseases. This bibliometric study examined developments in the field over the 2 years from the onset of the COVID-19 pandemic in early 2020 to August 2022 and completed an analysis of co-occurrence networks and keyword clustering by mapping collaborative networks of authors, countries, institutions, and journals through an analysis of published papers over this period. The studies that have contributed significantly to the field are highlighted, the available research results are summarized, and predictions and outlooks on future directions are presented. Given that the COVID-19 pandemic is still not over, research on stem cell therapies will continue, which in the long run will represent a disruptive change to the existing treatment paradigm.

## Data availability statement

The original contributions presented in the study are included in the article/supplementary material, further inquiries can be directed to the corresponding authors.

## Author contributions

XZ, JC, ZL, and YC participated in the conception and design of the study. JC and LC provided guidance on software for data and visual analysis. XZ retrieved and filtered the dataset from the Web of Science, completed the visual analysis of the data, and was a major contributor in writing the manuscript. QY, HT, JW, ZJ, and DZ were involved in the interpretation of the study findings. ZL, QY, JC, and LC modified the manuscript critically. All authors read and gave final approval for the submitted versions.

## Funding

This work was supported by the Special Fund Project of Guangdong Science and Technology (210728156901524, 210728156901519), Medical Scientific Research Foundation of Guangdong Province, China (Grant number A2021432), Shantou Medical Science and Technology Planning Project (Grant numbers 210521236491457, 210625106490696, 220518116490772, and 220518116490933), Administration of Traditional Chinese Medicine of Guangdong Province project (202205092315428030).

## Conflict of interest

The authors declare that the research was carried out independently of any commercial or financial relationships that might be considered conflicts of interest.

## Publisher's note

All claims expressed in this article are solely those of the authors and do not necessarily represent those of their affiliated organizations, or those of the publisher, the editors and the reviewers. Any product that may be evaluated in this article, or claim that may be made by its manufacturer, is not guaranteed or endorsed by the publisher.

## References

[1] Shih HI Wu CJ Tu YF Chi CY. Fighting COVID-19: a quick review of diagnoses, therapies, and vaccines. Biomed J. (2020) 43:341–54. 10.1016/j.bj.2020.05.021 32532623 PMC7260535

[2] World Health Organization. Weekly Epidemiological Update on COVID-19. Geneva: World Health Organization (2022). Available online at: https://www.who.int/publications/m/item/weekly-epidemiological-update-on-covid-19—24-august-2022 (accessed August 24, 2022).

[3] Gupta A Madhavan MV Sehgal K Nair N Mahajan S Sehrawat TS. Extrapulmonary manifestations of COVID-19. Nat Med. (2020) 26:1017–32. 10.1038/s41591-020-0968-3 32651579 PMC11972613

[4] Puntmann VO Carerj ML Wieters I Fahim M Arendt C Hoffmann J. Outcomes of cardiovascular magnetic resonance imaging in patients recently recovered from coronavirus disease 2019 (COVID-19). JAMA Cardiol. (2020) 5:1265–73. 10.1001/jamacardio.2020.3557 32730619 PMC7385689

[5] Topol EJ. COVID-19 can affect the heart. Science. (2020) 370:408–9. 10.1126/science.abe2813 32967937

[6] Xiong TY Redwood S Prendergast B Chen M. Coronaviruses and the cardiovascular system: acute and long-term implications. Eur Heart J. (2020) 41:1798–800. 10.1093/eurheartj/ehaa231 32186331 PMC7454513

[7] Paterson RW Brown RL Benjamin L Nortley R Wiethoff S Bharucha T. The emerging spectrum of COVID-19 neurology: clinical, radiological and laboratory findings. Brain. (2020) 143:3104–20. 10.1093/brain/awaa240 32637987 PMC7454352

[8] Wang D Hu B Hu C Zhu F Liu X Zhang J. Clinical characteristics of 138 hospitalized patients with 2019 novel coronavirus-infected pneumonia in Wuhan, China. JAMA. (2020) 323:1061–9. 10.1001/jama.2020.1585 32031570 PMC7042881

[9] Kuri-Cervantes L Pampena MB Meng W Rosenfeld AM Ittner CA Weisman AR. Comprehensive mapping of immune perturbations associated with severe COVID-19. Sci Immunol. (2020) 5:eabd7114. 10.1126/sciimmunol.abd7114 32669287 PMC7402634

[10] Riedel RN Pérez-Pérez A Sánchez-Margalet V Varone CL. Maymó JL. Stem cells and COVID-19: are the human amniotic cells a new hope for therapies against the SARS-CoV-2 virus? Stem Cell Res Ther. (2021) 12:155. 10.1186/s13287-021-02216-w 33648582 PMC7919997

[11] Yamanaka S. Pluripotent stem cell-based cell therapy-promise and challenges. Cell Stem Cell. (2020) 27:523–31. 10.1016/j.stem.2020.09.014 33007237

[12] Khatri M Richardson LA Meulia T. Mesenchymal stem cell-derived extracellular vesicles attenuate influenza virus-induced acute lung injury in a pig model. Stem Cell Res Ther. (2018) 9:17. 10.1186/s13287-018-0774-8 29378639 PMC5789598

[13] Regmi S Pathak S Kim JO Yong CS Jeong JH. Mesenchymal stem cell therapy for the treatment of inflammatory diseases: challenges, opportunities, and future perspectives. Eur J Cell Biol. (2019) 98:151041. 10.1016/j.ejcb.2019.04.002 31023504

[14] Cruz FF Borg ZD Goodwin M Sokocevic D Wagner DE Coffey A. Systemic administration of human bone marrow-derived mesenchymal stromal cell extracellular vesicles ameliorates aspergillus hyphal extract-induced allergic airway inflammation in immunocompetent mice. Stem Cells Transl Med. (2015) 4:1302–16. 10.5966/sctm.2014-0280 26378259 PMC4622402

[15] Gupta N Sinha R Krasnodembskaya A Xu X Nizet V Matthay MA. The TLR4-PAR1 axis regulates bone marrow mesenchymal stromal cell survival and therapeutic capacity in experimental bacterial pneumonia. Stem Cells. (2018) 36:796–806. 10.1002/stem.2796 29396891 PMC5918231

[16] Weiss DJ Segal K Casaburi R Hayes J Tashkin D. Effect of mesenchymal stromal cell infusions on lung function in COPD patients with high CRP levels. Respir Res. (2021) 22:142. 10.1186/s12931-021-01734-8 33964910 PMC8106850

[17] Fajgenbaum DC June CH. Cytokine storm. N Engl J Med. (2020) 383:2255–73. 10.1056/NEJMra2026131 33264547 PMC7727315

[18] Dhama K Patel SK Pathak M Yatoo MI Tiwari R Malik YS. An update on SARS-CoV-2/COVID-19 with particular reference to its clinical pathology, pathogenesis, immunopathology and mitigation strategies. Travel Med Infect Dis. (2020) 37:101755. 10.1016/j.tmaid.2020.101755 32479816 PMC7260597

[19] Uccelli A de Rosbo NK. The immunomodulatory function of mesenchymal stem cells: mode of action and pathways. Ann N Y Acad Sci. (2015) 1351:114–26. 10.1111/nyas.12815 26152292

[20] Lee JW Fang X Krasnodembskaya A Howard JP Matthay MA. Concise review: mesenchymal stem cells for acute lung injury: role of paracrine soluble factors. Stem Cells. (2011) 29:913–9. 10.1002/stem.643 21506195 PMC3293251

[21] Leng Z Zhu R Hou W Feng Y Yang Y Han Q. Transplantation of ACE2^−^ mesenchymal stem cells improves the outcome of patients with COVID-19 pneumonia. Aging Dis. (2020) 11:216–28. 10.14336/AD.2020.0228 32257537 PMC7069465

[22] Cargnoni A Romele P Bonassi Signoroni P Farigu S Magatti M Vertua E. Amniotic MSCs reduce pulmonary fibrosis by hampering lung B-cell recruitment, retention, and maturation. Stem Cells Transl Med. (2020) 9:1023–35. 10.1002/sctm.20-0068 32452646 PMC7445028

[23] Pritchard A. Statistical bibliography or bibliometrics. J Document. (1969) 25:348–9.

[24] Diem A Wolter SC. The use of bibliometrics to measure research performance in education sciences. Res High Educ. (2013) 54:86–114. 10.1007/s11162-012-9264-5

[25] Van Eck NJ Waltman L. Citation-based clustering of publications using CitNetExplorer and VOSviewer. Scientometrics. (2017) 111:1053–70. 10.1007/s11192-017-2300-7 28490825 PMC5400793

[26] Sevinc A. Web of science: a unique method of cited reference searching. J Natl Med Assoc. (2004) 96:980–3. 15253331 PMC2568431

[27] Sweileh WM Al-Jabi SW AbuTaha AS Zyoud SH Anayah FMA Sawalha AF. Bibliometric analysis of worldwide scientific literature in mobile health: 2006–2016. BMC Med Inform Decis Mak. (2017) 17:72. 10.1186/s12911-017-0476-7 28558687 PMC5450106

[28] He B Ding Y Ni C. Mining enriched contextual information of scientific collaboration: a meso perspective. J Am Soc Inform Sci Technol. (2011) 62:831–845. 10.1002/asi.21510

[29] Koseoglu MA Yildiz M Ciftci T. Authorship trends and collaboration patterns in business ethics literature. Bus Ethics A Eur Rev. (2018) 27:164–77. 10.1111/beer.12177

[30] van Eck NJ Waltman L. Software survey: VOSviewer, a computer program for bibliometric mapping. Scientometrics. (2010) 84:523–38. 10.1007/s11192-009-0146-3 20585380 PMC2883932

[31] Perianes-Rodriguez A Waltman L van Eck NJ. Constructing bibliometric networks: a comparison between full and fractional counting. J Inform. (2016) 10:1178–95. 10.1016/j.joi.2016.10.006

[32] Aria M Cuccurullo C. Bibliometrix: an R-tool for comprehensive science mapping analysis. J Inform. (2017) 11:959–75. 10.1016/j.joi.2017.08.007

[33] Hirsch JE. An index to quantify an individual's scientific research output. Proc Natl Acad Sci U S A. (2005) 102:16569–72. 10.1073/pnas.0507655102 16275915 PMC1283832

[34] Egghe L. Theory and practise of the g-index. Scientometrics. (2006) 69:131–52. 10.1007/s11192-006-0144-7

[35] Chen CM. CiteSpace II: detecting and visualizing emerging trends and transient patterns in scientific literature. J Am Soc Inform Sci Technol. (2006) 57:359–77. 10.1002/asi.20317

[36] Eyre-Walker A Stoletzki N. The assessment of science: the relative merits of post-publication review, the impact factor, and the number of citations. PLoS Biol. (2013) 11:e1001675. 10.1371/journal.pbio.1001675 24115908 PMC3792863

[37] Ye Q Wang B Mao J. The pathogenesis and treatment of the ‘Cytokine Storm' in COVID-19. J Infect. (2020) 80:607–13. 10.1016/j.jinf.2020.03.037 32283152 PMC7194613

[38] Kox M Waalders NJB Kooistra EJ Gerretsen J Pickkers P. Cytokine levels in critically ill patients with COVID-19 and other conditions. JAMA. (2020) 324:1565–7. 10.1001/jama.2020.17052 32880615 PMC7489366

[39] Samavati L Uhal BD. ACE2, much more than just a receptor for SARS-CoV-2. Front Cell Infect Microbiol. (2020) 10:317. 10.3389/fcimb.2020.00317 32582574 PMC7294848

[40] Rad AE Brinjikji W Cloft HJ Kallmes DF. The H-index in academic radiology. Acad Radiol. (2010) 17:817–21. 10.1016/j.acra.2010.03.011 20471868

[41] Nishiga M Wang DW Han Y Lewis DB Wu JC. COVID-19 and cardiovascular disease: from basic mechanisms to clinical perspectives. Nat Rev Cardiol. (2020) 17:543–58. 10.1038/s41569-020-0413-9 32690910 PMC7370876

[42] Huang C Wang Y Li X Ren L Zhao J Hu Y. Clinical features of patients infected with 2019 novel coronavirus in Wuhan, China. Lancet. (2020) 395:497–506. 10.1016/S0140-6736(20)30183-5 31986264 PMC7159299

[43] Hoffmann M Kleine-Weber H Schroeder S Krüger N Herrler T Erichsen S. SARS-CoV-2 cell entry depends on ACE2 and TMPRSS2 and is blocked by a clinically proven protease inhibitor. Cell. (2020) 181:271–80.e8. 10.1016/j.cell.2020.02.052 32142651 PMC7102627

[44] Sengupta V Sengupta S Lazo A Woods P Nolan A Bremer N. Exosomes derived from bone marrow mesenchymal stem cells as treatment for severe COVID-19. Stem Cells Dev. (2020) 29:747–54. 10.1089/scd.2020.0080 32380908 PMC7310206

[45] Xu Z Shi L Wang Y Zhang J Huang L Zhang C. Pathological findings of COVID-19 associated with acute respiratory distress syndrome. Lancet Respir Med. (2020) 8:420–2. 10.1016/S2213-2600(20)30076-X 32085846 PMC7164771

[46] Wilson JG Liu KD Zhuo H Caballero L McMillan M Fang X. Mesenchymal stem (stromal) cells for treatment of ARDS: a phase 1 clinical trial. Lancet Respir Med. (2015) 3:24–32. 10.1016/S2213-2600(14)70291-7 25529339 PMC4297579

[47] Metcalfe SM. Mesenchymal stem cells and management of COVID-19 pneumonia. Med Drug Discov. (2020) 5:100019. 10.1016/j.medidd.2020.100019 32296777 PMC7147223

[48] Zhang J Yu Q Zheng FS Long C Lu ZX Duan ZG. Comparing keywords plus of WOS and author keywords: a case study of patient adherence research. J Assoc Inform Sci Technol. (2016) 67:967–72. 10.1002/asi.23437

[49] Yen BL Yen ML Wang LT Liu KJ Sytwu HK. Current status of mesenchymal stem cell therapy for immune/inflammatory lung disorders: gleaning insights for possible use in COVID-19. Stem Cells Transl Med. (2020) 9:1163–73. 10.1002/sctm.20-0186 32526079 PMC7300965

[50] Li H Liu SM Yu XH Tang SL Tang CK. Coronavirus disease 2019 (COVID-19): current status and future perspectives. Int J Antimicrob Agents. (2020) 55:105951. 10.1016/j.ijantimicag.2020.105951 32234466 PMC7139247

[51] Liu B Li M Zhou Z Guan X Xiang Y. Can we use interleukin-6 (IL-6) blockade for coronavirus disease 2019 (COVID-19)-induced cytokine release syndrome (CRS)? J Autoimmun. (2020) 111:102452. 10.1016/j.jaut.2020.102452 32291137 PMC7151347

[52] Riva L Yuan S Yin X Martin-Sancho L Matsunaga N Pache L. Discovery of SARS-CoV-2 antiviral drugs through large-scale compound repurposing. Nature. (2020) 586:113–9. 10.1038/s41586-020-2577-1 32707573 PMC7603405

[53] Wang J Jiang M Chen X Montaner LJ. Cytokine storm and leukocyte changes in mild vs. severe SARS-CoV-2 infection: review of 3939 COVID-19 patients in China and emerging pathogenesis and therapy concepts. J Leukoc Biol. (2020) 108:17–41. 10.1002/JLB.3COVR0520-272R 32534467 PMC7323250

[54] Lotfi M Hamblin MR Rezaei N. COVID-19: transmission, prevention, and potential therapeutic opportunities. Clin Chim Acta. (2020) 508:254–66. 10.1016/j.cca.2020.05.044 32474009 PMC7256510

[55] Ulrich H Pillat MM. CD147 as a target for COVID-19 treatment: suggested effects of azithromycin and stem cell engagement. Stem Cell Rev Rep. (2020) 16:434–40. 10.1007/s12015-020-09976-7 32307653 PMC7167302

[56] Li Z Niu S Guo B Gao T Wang L Wang Y. Stem cell therapy for COVID-19, ARDS and pulmonary fibrosis. Cell Prolif. (2020) 53:e12939. 10.1111/cpr.12939 33098357 PMC7645923

[57] Wick KD Leligdowicz A Zhuo H Ware LB Matthay MA. Mesenchymal stromal cells reduce evidence of lung injury in patients with ARDS. JCI Insight. (2021) 6:e148983. 10.1172/jci.insight.148983 33974564 PMC8262503

[58] Ji HL Liu C Zhao RZ. Stem cell therapy for COVID-19 and other respiratory diseases: global trends of clinical trials. World J Stem Cells. (2020) 12:471–80. 10.4252/wjsc.v12.i6.471 32742564 PMC7360994

[59] Lanzoni G Linetsky E Correa D Messinger Cayetano S Alvarez RA Kouroupis D. Umbilical cord mesenchymal stem cells for COVID-19 acute respiratory distress syndrome: a double-blind, phase 1/2a, randomized controlled trial. Stem Cells Transl Med. (2021) 10:660–73. 10.1002/sctm.20-0472 33400390 PMC8046040

[60] Fischer UM Harting MT Jimenez F Monzon-Posadas WO Xue H Savitz SI. Pulmonary passage is a major obstacle for intravenous stem cell delivery: the pulmonary first-pass effect. Stem Cells Dev. (2009) 18:683–92. 10.1089/scd.2008.0253 19099374 PMC3190292

[61] Papazian L Aubron C Brochard L Chiche JD Combes A Dreyfuss D. Formal guidelines: management of acute respiratory distress syndrome. Ann Intens Care. (2019) 9:69. 10.1186/s13613-019-0540-9 31197492 PMC6565761

